# Epitope-coated polymer particles elicit neutralising antibodies against *Plasmodium falciparum* sporozoites

**DOI:** 10.1038/s41541-021-00408-2

**Published:** 2021-11-29

**Authors:** Benjamin J. Evert, Shuxiong Chen, Robyn McConville, Ryan W. J. Steel, Julie Healer, Justin A. Boddey, Lucas Huntimer, Bernd H. A. Rehm

**Affiliations:** 1 Centre for Cell Factories and Biopolymers, Griffith Institute for Drug Discovery, Griffith University, Brisbane, QLD Australia; 2 The Walter and Eliza Hall Institute of Medical Research, Parkville, VIC Australia; 3 Department of Medical Biology, The University of Melbourne, Parkville, VIC Australia; 4 Elanco, Indianapolis, IN USA; 5 Menzies Health Institute Queensland (MHIQ), Griffith University, Gold Coast, QLD Australia

**Keywords:** Peptide vaccines, Malaria

## Abstract

The current Malaria RTS,S vaccine is based on virus-like particles (VLPs) comprising the NANP repetitive epitopes from the cicumsporozoite protein (CSP) of *Plasmodium falciparum*. This vaccine has limited efficacy, only preventing severe disease in about 30% of vaccinated individuals. A more efficacious vaccine is urgently needed to combat malaria. Here we developed a particulate malaria vaccine based on the same CSP epitopes but using biopolymer particles (BPs) as an antigen carrier system. Specific B- and T-cell epitope-coated BPs were assembled in vivo inside an engineered endotoxin-free mutant of *Escherichia coli*. A high-yield production process leading to ~27% BP vaccine weight over biomass was established. The epitope-coated BPs were purified and their composition, i.e., the polymer core and epitope identity, was confirmed. Epitope-coated BPs were used alongside soluble peptide epitopes and empty BPs to vaccinate sheep. Epitope-coated BPs showed enhanced immunogenicity by inducing anti-NANP antibody titre of EC50 > 150,000 that were at least 20 times higher than induced by the soluble peptides. We concluded that the additional T-cell epitope was not required as it did not enhance immunogenicity when compared with the B-cell epitope-coated BPs. Antibodies specifically bound to the surface of *Plasmodium falciparum* sporozoites and efficiently inhibited sporozoite motility and traversal of human hepatocytes. This study demonstrated the utility of biologically self-assembled epitope-coated BPs as an epitope carrier for inclusion in next-generation malaria vaccines.

## Introduction

Malaria is a disease caused by the *Plasmodium* parasite, predominantly *P. falciparum*, which is transmitted through the bites of infected mosquitoes. It is arguably one of the world’s most devastating diseases, with ~228 million cases and 405 thousand deaths annually, most of which are of children in developing countries^1^. Despite the introduction of the first commercially available malaria vaccine, RTS,S, the fight against malaria appears to have stalled, with only very slight reduction in cases seen over the past few years^1^. The main contributing factors for this are the fact that the RTS,S vaccine shows only about 30% efficacy and drug resistance among parasites and parasite vectors is becoming more widespread^2–4^. This highlights that a more effective malaria vaccine is urgently needed. As malaria is prevalent in developing countries, the vaccine would ideally be stable at ambient temperatures avoiding cold-chain requirements and it would be subject to cost-effective manufacture. The development of such a vaccine provides the best chance of combating the disease.

There are three main strategies for malaria vaccines; transmission blocking vaccines, pre-erythrocytic vaccines and blood-stage vaccines, which target either the mosquito, liver, or blood stage of the parasite’s lifecycle, respectively. The current RTS,S vaccine adopts the pre-erythrocytic strategy based on the *P. falciparum* circumsporozoite protein (CSP) expressed on the surface of sporozoites^5^. An important feature of CSP is a repetitive region that contains 25 to 42 copies of the amino acids NANP^6^. Antibodies against this repetitive portion of CSP play a vital role in developing protective immunity^5^. Briefly the RTS,S vaccine contains 19 copies of the NANP repeats as well as an important C-terminal type 1 thrombospondin repeat (TSR) region of CSP fused to the N terminus of the hepatitis B S envelope protein, which forms virus-like particles that display the CSP antigens^5^. Notably the NANP repeat region lacks T-cell epitopes and induces little or no T-cell response and the TSR domain antigen is not glycosylated like the endogenous protein. These may be important to consider when developing future malaria vaccines^7–10^. The RTS,S vaccine was proposed in 1995, since then several other vaccines have been developed also based on the CSP repeat region but include additional newly identified T-cell epitopes, with the aim of improving the current RTS,S vaccine^11–20^. One of these vaccines, which has currently completed phase 2b clinical trials, is the R21 vaccine. The R21 vaccine also utilises the hepatitis B virus as a platform to display the repetitive NANP_3_ epitope^21^. The major difference is that the RTS,S vaccine uses a mix of unmodified S protein and S-CSP fusion protein whereas the R21 uses only the S-CSP fusion protein resulting in the formation of VLPs that contain a much higher proportion of NANP_3_^21^.

The present study uses biopolymer particles (BPs) as an antigen delivery system to elicit strong and functional antibody responses against repetitive B- and B/T-cell epitopes from the CSP antigen to mediate protective immunity. It aims to utilise the BP vaccine platform’s unique advantages such as cost-effective scalable production and ambient-temperature stability of the vaccine product. Peptide vaccines have a long history in malaria vaccine development, but presentation on a particle offers the advantage of much improved immunogenicity^22–27^.

The BP technology is a newly developed vaccine platform based on the ability to engineer *E. coli* to produce spherical antigen-coated biopolymer inclusions^28^. The BP approach is distinct from other polymer particle-based vaccine approaches offering major advantages regarding safety, efficacy and manufacturability^29^. The bacterial PHB synthase (PhaC) mediates assembly of spherical BP inclusions. PhaC remains covalently attached at the surface and can be exploited as a BP anchoring domain. Structure-function analysis and protein engineering enabled the identification of permissive fusion points and insertion sites for incorporating foreign proteins^30,31^. Introduction of a hybrid gene encoding a PhaC fusion protein including selected antigens results in high-yield production of antigen-coated BPs contributing to up to 70% of biomass in recombinant *E. coli*^32–36^. Previously antigen-coated BPs have been developed and subjected to several animal trials showing safety and induction of specific cell-mediated (Th1, Th2 and Th17) and humoral immune responses associated with protective immunity against pathogens such as *Mycobacterium tuberculosis*^37^, *Streptococcus pneumonia*^38^, *Neisseria meningitidis*^39^, HCV^35^ and SARS-CoV-2^40^. The BPs were safe, as all vaccinated animals remained healthy, gained weight normally and showed no abnormal behaviour. The biopolymer itself is a natural biopolyester, which is composed of polyhydroxybutyrate (PHB), a compound also found in human blood^41^. PHB has been approved by the FDA as implant material for clinical trials in humans^42^. Our preferred production host is an endotoxin-free mutant of *E. coli* (ClearColi^TM^, Lucigen). *E. coli* is generally recognised as a safe (GRAS) production host and is the preferred host for producing numerous therapeutic proteins. Importantly, the PHB synthase, which is derived from the safe soil bacterium *Ralstonia eutropha*, was experimentally shown not to induce a detectable immune response, i.e., is not or poorly immunogenic^43^. All these properties illustrate the suitability of BPs as a platform for malaria vaccine development. Hence combining the tested epitopes from the RTS,S vaccine and newly identified CSP T-cell epitopes with the BP technology might provide an effective strategy for the development of a malaria vaccine candidate.

**Fig. 1:**
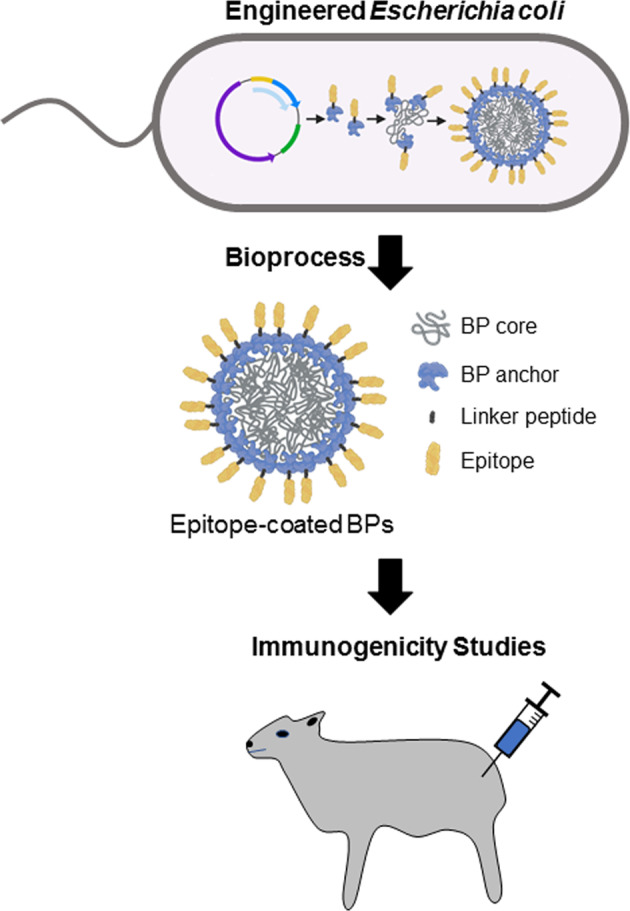
Schematic of BP vaccine production and composition. An endotoxin-free production strain of E. coli, ClearColi BL21(DE3), was bioengineered to produce *P. falciparum* CSP epitope-coated BPs in one step. Immunogenicity of epitope-coated BPs was analysed in the sheep model.

Here, we designed two BPs coated with either B- or B/T-cell epitope repeats from *P. falciparum* CSP guided by the design of the RTS,S, R21 and other experimental malaria vaccines (Fig. 1). We performed animal trials in sheep and evaluated functional immune responses against *P. falciparum* sporozoites after vaccination. The sheep model was used to allow better translation of vaccine efficacy toward human uses.

## Results

### Design, production, and characterisation of BPs

**Fig. 2:**
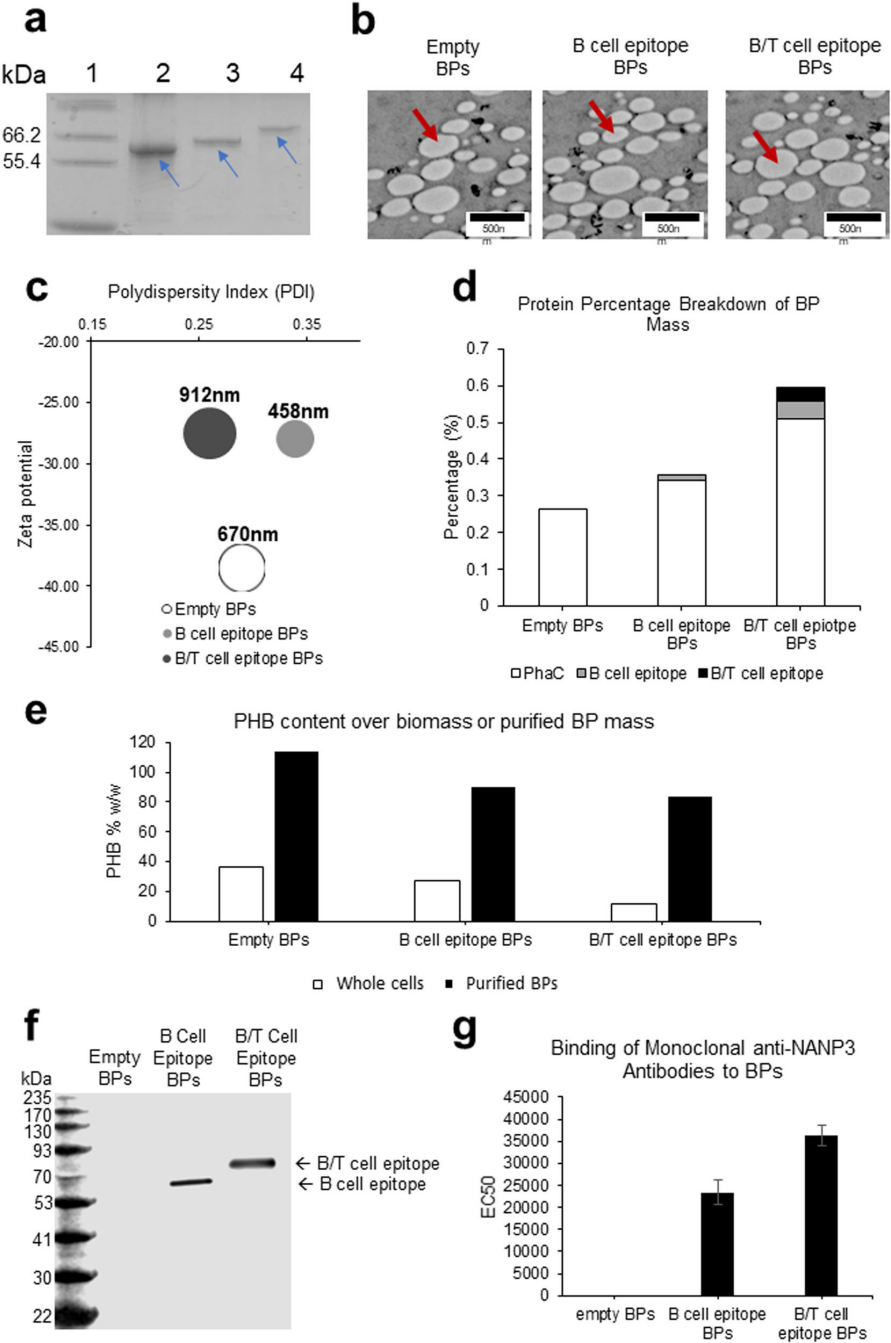
Characterisation of the BP vaccines. **a** Analysis of BP-associated proteins using SDS-PAGE indicated by blue arrows. Lane 1, Molecular weight standard (Mark12™ Unstained protein standard); Lane 2, Empty BPs; Lane 3, B-cell epitope BPs; Lane 4, B/T-cell epitope BPs. Blue arrows indicate respective full-length fusion protein corresponding with their expected molecular weight. **b** TEM images of the BPs. Individual BPs are indicated by red arrows. **c** Bubble graph showing properties of BPs. Polydispersity index (PDI) is shown along the *x*-axis, Zeta potential is shown along the *y*-axis, while size of bubbles indicates average BP size. All data were collected from three technical replicates. **d** Graph showing protein proportion of total BP mass determined by densitometry using Image J and known BSA standards. **e** Graph showing % w/w of PHB contributing to whole-cell mass or purified BP mass as determined by HPLC. **f** Immunoblot analysis to assess specific recognition of B- and B/T-cell epitopes presented by BPs using the monoclonal anti-NANP_3_ antibody. **g** Average EC50 titre of the monoclonal antibody binding to B- and B/T-cell epitope-coated BPs. Statistical significance was determined by Nonparametric Mann–Whitney (Minitab 19) when *p* < 0.05. BPs used for the animal trial were analysed using three replicates. All EC50 values were statistically significant from one another. *p* = 0.007 for empty BPs compared with B-cell epitope BPs. *p* = 0.002 for Empty BPs compared with B/T-cell epitope BPs. *p* = 0.03 for B-cell epitope BPs compared with B/T-cell epitope BPs. All data are mean and error bars represent +/–SD.

To generate antigen-coated BPs we designed two synthetic genes, encoding the BP anchoring domain (PhaC) fused to two repeats of malaria epitopes (B-cell epitope—Amino Acid Sequence: NANPNANPNANP; B-cell epitope flanked by T-cell epitopes—Amino Acid Sequence: DPNANPNVDPNANPNVNANPNANPNANPEYLNKIQ NSLSTEWSPCSVT (Supplementary Table 1). Overexpression plasmids were generated harbouring genes encoding PhaC, PhaC-B-cell epitope fusion protein and PhaC-B/T-cell epitope fusion protein under control of the strong T7 promoter. Plasmids with only PhaC were used to produce empty BPs to serve as antigen carrier control. These plasmids were transformed into engineered *E. coli* that produces the PHB precursor, (*R*)−3-hydroxybutyryl-CoA and were expressed in *E. coli*, grown in 2 L bioreactors using animal component-free synthetic media (Fig. 1). All full-length fusion proteins were produced and mediated the assembly of BPs indicating retention of PhaC function within the fusion proteins (Fig. 2).

To assess BP-associated proteins, we subjected BPs to sodium dodecyl sulfate−polyacrylamide gel electrophoresis (SDS-PAGE) analysis, which showed proteins corresponding to theoretical molecular weights of the full-length proteins composed of the PhaC anchoring domain and respective malaria epitopes (Fig. 2a). TEM images confirmed the presence of spherically shaped BPs after isolation from the *E. coli* production strains and which resembled the appearance of previously isolated BPs (Fig. 2b)^39,44^. BP size, charge and polydispersity were analysed (Fig. 2c) and were similar to previously produced surface-coated BPs^44–49^ suggesting structural integrity of BPs and suitability for vaccination. All polydispersity indexes were <35% suggesting suitability for clinical applications^50^. Average BP size was found to be slightly different for each BP variant, but all had anionic surface charges (Fig. 2c). Densitometry analysis revealed the fusion protein percentage over BP mass and subsequently the antigen percentage of the BP mass (Fig. 2d). High-performance liquid chromatography (HPLC) was performed to determine the PHB content of whole cells producing BPs and of purified BPs. The PHB % of dry weight for whole cells expressing PhaC, PhaC-B-cell epitope and PhaC-B/T-cell epitope was 36.7%, 26.9% and 11.7% respectively (Fig. 2e). The PHB % of dry weight for purified BP samples was about 114%, 90% and 84%, respectively (Fig. 2e). Furthermore, we obtained the monoclonal anti-NANP3 antibody (clone 2A10) from BEI resources and showed specific binding to epitope-coated BPs in enzyme-linked immunosorbent assay (ELISA) and to the fusion proteins in immunoblots (Fig. 2f, g). ELISA EC50 titre were deduced^33^ and led to average EC50 values of about 23,320 and 36,442 for the B-cell epitope-coated BPs and B/T-cell epitope-coated BPs, respectively, suggesting high density and surface exposure of NANP_3_ peptide attached to BPs (Fig. 2f). Immunoblots showed that the antibody binding was specific for the B- and B/T-cell epitope fusion protein. No binding was seen for the PhaC carrier protein on the empty BPs (Fig. 2g).

This confirms that the epitope is surface exposed, i.e. accessible by the antibody in ELISA, while further confirming that the epitope is part of the fusion protein and retains its antigenicity. The BP-associated proteins were digested with trypsin and the resulting peptides were separated by liquid chromatography. Peptides masses were determined by matrix-assisted laser desorption ionisation-time of flight mass spectrometry (MALDI-TOF/MS) enabling identification of the respective full-length fusion proteins. Identified peptides of the BP-associated fusion proteins are indicated as highlighted in green (Supplementary Table 2). In the case of the B-cell epitope comprising fusion protein no tryptic peptide was obtained within the epitope region, hence N terminal amino acid sequencing was applied, which provided evidence for the identity of the fusion protein (Sequenced N terminus: MNANXN(D/A)NPD).

### B- and B/T-cell epitope-coated BPs induced a strong and specific immune response

**Fig. 3:**
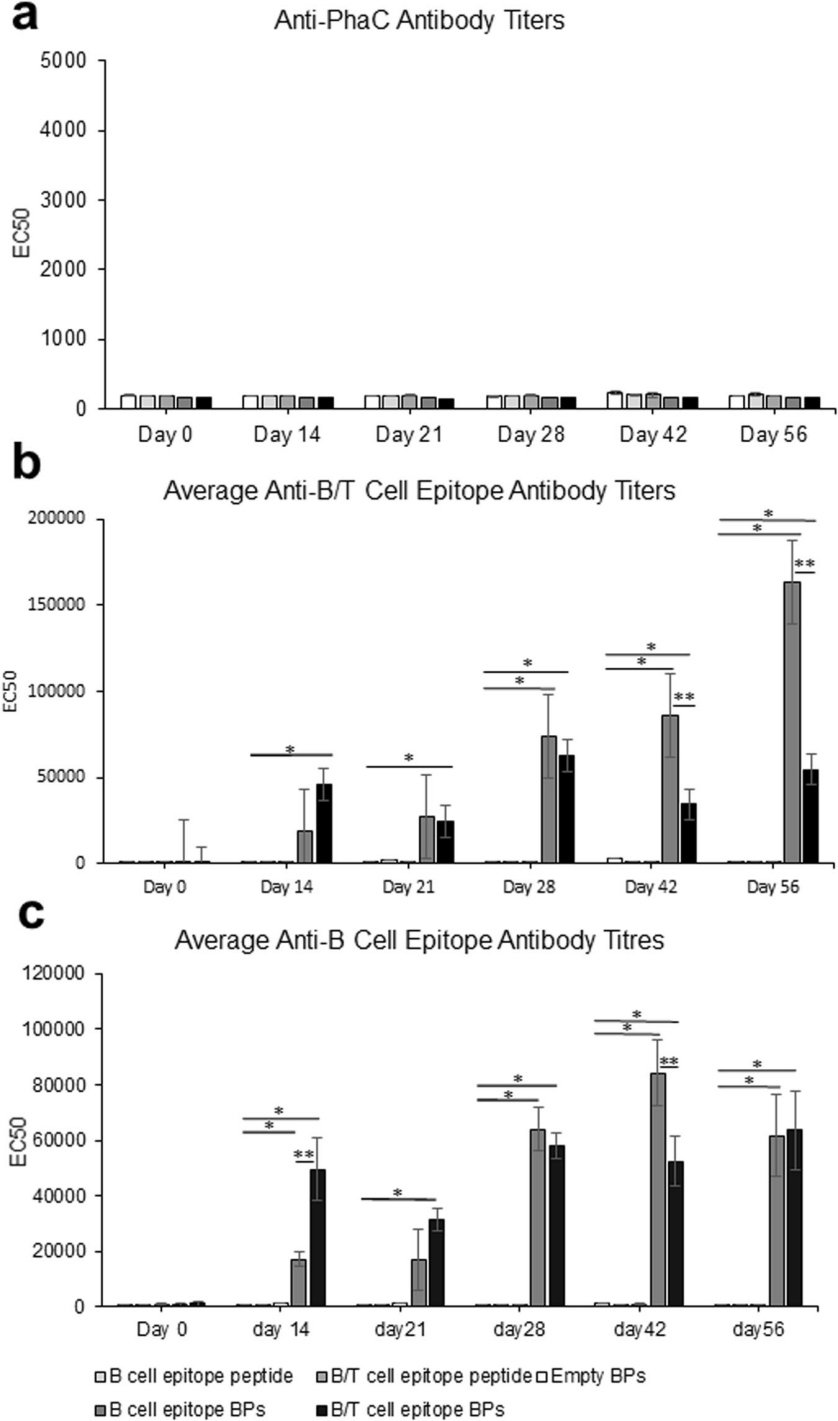
Antibody responses presented as EC50 values determined by ELISA. *Sheep immunised with either empty BPs indicated by white, B-cell epitope peptide indicated by light grey, B/T-cell epitope peptide indicated by grey, B-cell epitope-coated BPs indicated by dark grey and B/T-cell epitope-coated* BPs indicated by black. Statistical significance was determined by Nonparametric Mann–Whitney (Minitab 19) when *p* < 0.05. * indicates statistical significance compared to empty BPs and epitope peptides. ** indicates statistical significance between B- and B/T-cell epitope-coated BPs. Eight individual sheep serum samples per group were analysed as each three technical replicates. **a** shows antibody titre representing binding to empty BPs quantified by ELISA. **b** shows antibody titre representing binding to B-cell epitope-coated BPs quantified by ELISA. **c** shows antibody titre representing binding to B/T-cell epitope-coated BPs quantified by ELISA. All data are mean and error bars represent +/–SD.

Purified BPs coated with B- or B/T-cell epitopes derived from CSP or respective soluble epitope peptides were formulated in the water in oil adjuvant XStend III (56% (v/v) in 10 mM Tris buffer pH 7.5) using homogenisation. This adjuvant was selected as it is approved for veterinary applications and resembles the adjuvant MF59 approved for human uses. Eight sheep per group were either vaccinated with 25 µg of B-cell epitopes on BPs or 50 µg B/T-cell epitopes on BPs or same BP mass of empty BPs or 74 µg synthetic peptide NANP_3_ (B-cell epitope) or 74 µg of synthetic peptides NANP_3_ (B-cell epitope) mixed with EYLNKIQNSLSTEWSPCSVT (T-cell epitope) per dose. The amount B/T epitopes attached to BPs per dose was doubled compared to only B epitopes attached to BPs in order to inject similar copy numbers of the B-cell epitope. The dose was chosen guided by antigen amounts used in subunit vaccines licensed for human uses. Vaccines were administered subcutaneously adhering to a prime-boost-boost (14d apart) immunisation protocol. ELISAs were used to quantify antibodies against the B- and B/T-cell epitopes as induced by the empty BPs (PhaC), B-cell epitope peptide, B/T-cell epitope peptide, B-cell epitope BPs and B/T-epitope BPs (Fig. 3). The ELISA results showed induction of strong and specific antibody responses to the B/T-cell epitope for B- and B/T-cell epitope-coated BP vaccinated groups reaching maximum average EC50 titre of ~163,000 and ~55,000, respectively, after the second boost. Strong responses to the B-cell epitope amounted to maximum average EC50 titer of ~84,000 and ~64,000 for the groups vaccinated with B-cell epitope-coated or B/T-cell epitope-coated BPs, respectively. Maximum average EC50 values of <790 were found for the group vaccinated with empty BPs. These data suggested that the empty BPs themselves did not induce significant levels of antibodies that could recognise B- or T-cell epitopes. The soluble synthetic peptides did not induce significant immune responses as indicated by maximum average EC50 values of 1278 and 3035 for B- and B/T-cell peptides, respectively. These data suggest that the display of peptides on BPs strongly boosts the immunogenicity of these epitopes, which was previously observed for other antigens in the context of bacterial and viral pathogens^51^. It is also evident that the T-cell epitope is not required to induce strong antibody responses to the B-cell epitope. None of the groups produced significant antibody titre to the BP carrier, i.e., empty BPs, suggesting no carrier suppression occurred.

**Fig. 4:**
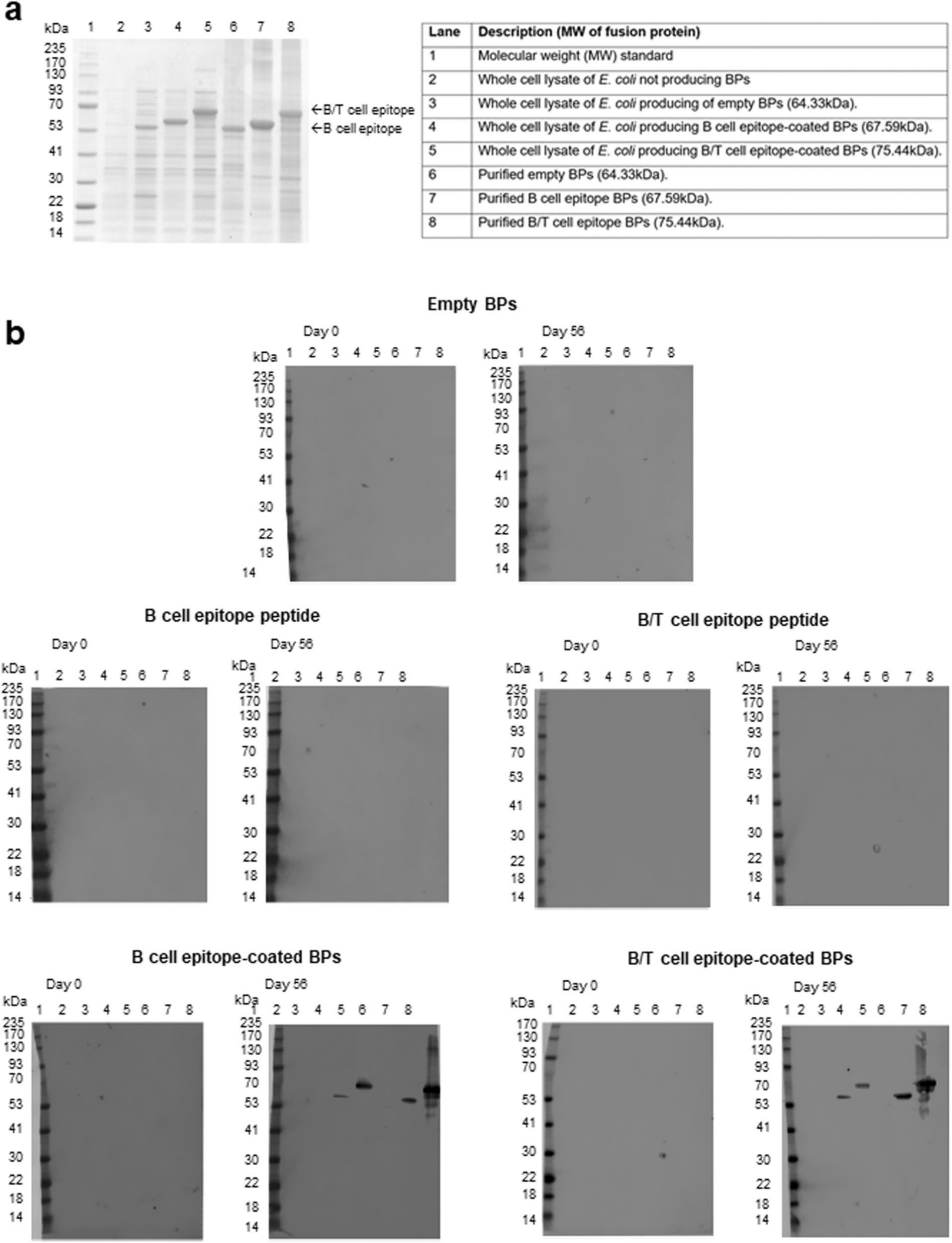
Analysis of specificity of immune response. Immunoblot analysis to assess specific recognition of B- and B/T-cell epitopes (malaria CS-derived NANP repeats) by antibodies in pooled sera from vaccinated sheep **a** SDS-PAGE analysis of E. coli whole-cell lysates and purified BPs used for immunoblotting. **b** shows immunoblotting results using pooled sera purified from vaccination group on day 0 (pre-immune) and day 56 (post vaccination).

To assess whether immune responses were specific to the displayed epitopes, we performed immunoblotting of pooled sera (pre-immune sera and sera after the third vaccination) against whole-cell lysates of the *E. coli* production strain and purified BP fractions separated by SDS-PAGE. The immunoblotting results clearly indicated induction of highly specific antibody responses to the B- and B/T-cell epitope in groups vaccinated with epitope-coated BPs (Fig. 4). Neither responses to the BP anchoring domain (PhaC) nor to potential host cell protein impurities were detectable.

### Serum-derived IgG bound to *P. falciparum* sporozoites and inhibited traversal of human hepatocytes

**Fig. 5:**
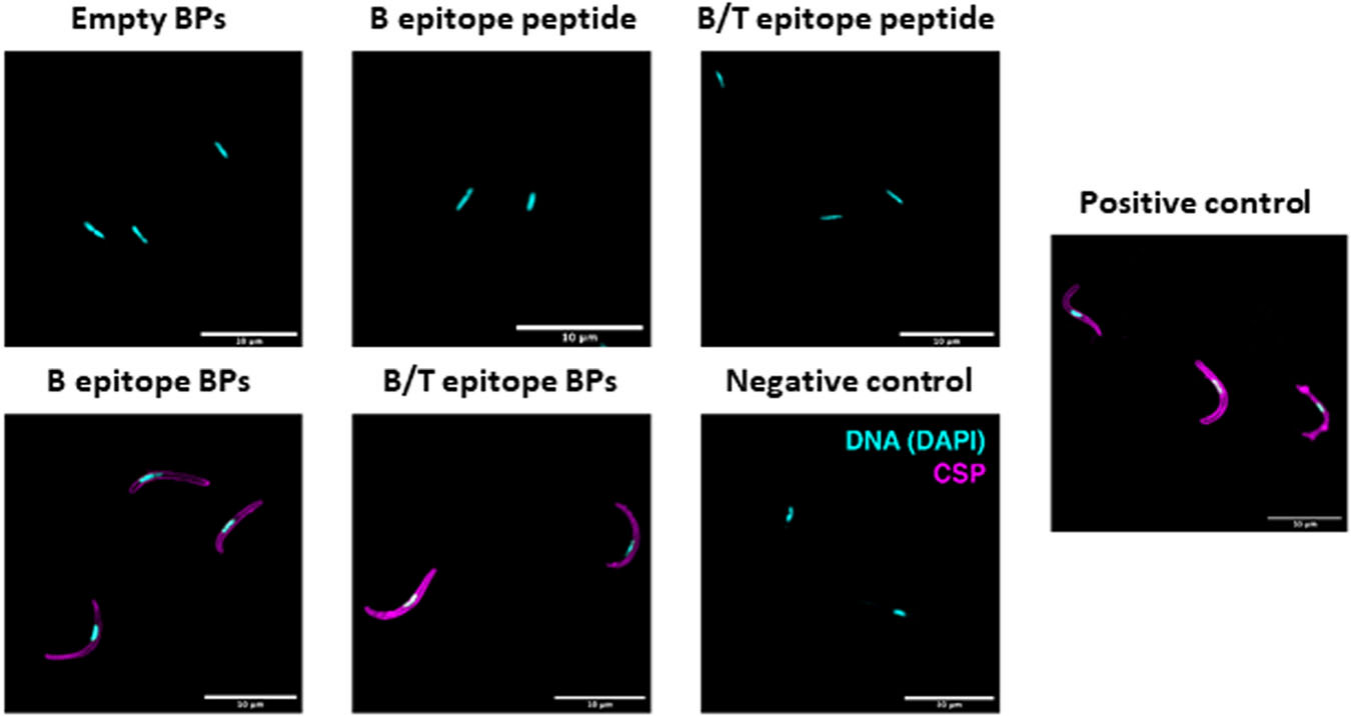
Binding of antibodies from vaccinated sheep to *P. falciparum* NF54 sporozoites. Sporozoites labelled with antibodies purified from sheep injected with the different vaccines. Antibodies from sheep vaccinated with the soluble B- and B/T-cell epitope peptides and the empty BPs did not label the sporozoite surface like the monoclonal anti-NANP3 antibody (CSP; clone 2A10) that served as positive control (purple). Antibodies from sheep vaccinated with the B- and B/T-cell epitope-coated BPs labelled the sporozoite surface (purple). Empty BPs and only the anti-sheep antibody (negative controls) served as control and did not surface label sporozoites. DNA was visualised with DAPI (magenta). Scale bars, 10 µm.

To determine antibody functionality, we purified IgG from sheep serum using protein G-coated BPs^52^. Purified antibodies from each group were incubated with fixed *P. falciparum* salivary gland sporozoites and bound antibodies were detected with secondary anti-sheep IgG-Alexa488 antibody conjugate as a fluorescent secondary antibody by fluorescent microscopy (Fig. 5).

**Fig. 6:**
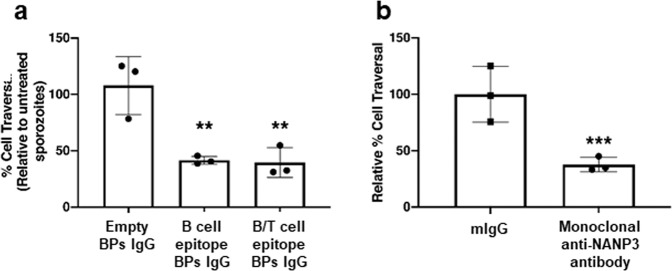
Antibodies from vaccinated sheep bind *P. falciparum* sporozoites and inhibit traversal through hepatocytes. **a** HC-04 cell traversal by sporozoites in the presence of antibodies from sheep vaccinated with empty BP, B-cell epitope-coated BPs and B/T-cell epitope-coated BPs, respectively. **b** HC-04 cell traversal by sporozoites in the presence naïve mouse IgG (mIgG) or monoclonal anti-NANP3 (clone 2A10) served as negative and positive control, respectively. For gating strategy see Supplementary Material Fig. 1. Data shown are pooled from three independent experiments relative to the no sporozoites control. *p* < 0.01 using Wilcoxon test. ** indicates statistical significance from empty BPs. *** indicates statistical significance from the mouse IgG negative control. All antibodies were used at 10 μg/ml. All data are mean and error bars represent +/–SD.

We demonstrated that IgG purified from serum of sheep injected with B- and B/T-cell epitope-coated BPs, respectively, were able to bind *P. falciparum* sporozoites (Fig. 5). Meanwhile IgG purified from serum of sheep injected with B-cell epitope peptide, B/T-cell epitope peptide and empty BPs, respectively, showed no binding (Fig. 5). Based on these results we selected IgG derived from serum of sheep injected with empty, B cell and B/T-cell BPs for further functional analysis to determine if these antibodies could inhibit sporozoite traversal of human hepatocytes, which requires gliding motility, using a standard cell traversal assay^53^. Human HC-04 cells were mixed with motile *P. falciparum* NF54 wildtype sporozoites and incubated together in cell culture medium containing cell-impermeable Dextran (MW of 10,000) conjugated to FITC and either 10 µg/ml IgG derived from sheep serum, 10 µg/ml mouse IgG as a negative control or 10 µg/ml anti-PfCSP mAb 2A10 as a positive control. Upon traversal by sporozoites, wounding and resealing of the HC-04 cells permitted entry of dextran into the cells and this was quantified by fluorescence activated cell sorting (FACS) to determine the relative % traversal of HC-04 cells by motile sporozoites. This method was first validated using uninfected cells, 10 µg/ml mouse IgG as a negative control and 10 µg/ml anti-PfCSP mAb 2A10 as a positive control (Supplementary Fig. 1). We confirmed that unspecific mouse IgG had no inhibition, whereas the anti-PfCSP mAb2A10 inhibited sporozoite traversal. The same experiment was performed using IgG purified from sheep serum (Fig. 6a). IgG purified from sheep injected with B- or B/T-cell epitope-coated BPs significantly reduced cell traversal compared to control IgG purified from serum of sheep injected with empty BPs, and the inhibition was comparable to the positive control 2A10 anti-CSP antibody that inhibits motility and functional cell traversal (Fig. 6b). Collectively, this showed that immunisation with B- and B/T-cell epitope-coated BPs produced functionally inhibitory antibodies against *P. falciparum* sporozoites.

## Discussion

There is a world-wide interest in developing a safe and efficient malaria vaccine that shows improved performance over the current RTS,S vaccine. Here, we used an innovative approach of assembling malaria epitope-coated BPs inside engineered *E. coli* to not only produce a safe and efficient vaccine candidate but to also provide means for cost-effective large-scale manufacture using the well-established industrial workhorse *E. coli*. We previously showed that antigen-coated BPs can be manufactured at >50% of biomass as well as that they can serve as efficient antigen carrier in particulate vaccine formulation by demonstrating induction of protective immunity against bacterial and viral pathogens in animal models^32–36^. Here, we used a bioengineering approach that employs *E. coli* as a cell factory to assemble CSP-derived B- or B/T-cell epitope-coated BPs (Fig. 1, Supplementary Table 1). We chose the NANP_3_ peptide for the B-cell epitope-coated BPs, which is used in the licensed RTS,S. We chose the previously identified universal T-cell epitopes DPNANPNVDPNANPNV and EYLNKIQNSLSTEWSPCSVT peptides flanking the NANP_3_ repeat region for the B/T-cell epitope BPs^54^. These T-cell epitopes were originally identified by CD4^+^-T-cell clones isolated from *P. falciparum* sporozoite-immunised persons^55,56^.

We first evaluated the potential of these epitope-coated BPs as particulate malaria vaccine by assessing their characteristics. BPs were confirmed to be coated with the correct B- and B/T-cell epitope-PhaC fusion proteins by SDS-PAGE analysis, MALDI-TOF, and N terminal sequencing (Fig. 2a and Supplementary Table 2). TEM confirmed the expected spherical shape of the BPs (Fig. 2b). BP formation itself suggested the fusion of the B- and B/T-cell epitopes to the BP anchoring PhaC protein did not impact on its ability to catalyse polymer synthesis and to mediate self-assembly of BPs. Size analysis revealed that all BP samples had a polydispersity index (PDI) of < 35% (Fig. 2c). Generally, particles with PDI < 30% are considered suitable for clinical use^50^. The observed differences in BP sizes (Fig. 2c) are consistent with previous studies, where translational fusion of different antigens/epitopes had impacted on BP size^32–36^. These differences had no obvious impact on immunogenicity.

Densitometry analysis of BP fusion proteins showed that both B- and B/T-cell epitope-coated BPs had higher protein amount over BP mass compared to empty BPs with B/T-cell epitope-coated BPs showing the highest protein amount (Fig. 2d). Overall analysis of the BPs confirms the expected characteristics and highlights the suitability for vaccine application.

HPLC of whole-cell samples after expression of BPs in bioreactors showed a PHB content (% w/w) of about 37%, 27% and 12% for *E coli* cells producing empty BPs, B-cell epitope BPs and B/T-cell epitope BPs respectively (Fig. 2e). Previously we have been able to achieve > 50% in shake flask expression cultures^32–36^. We expect bioreactor-based production can be further optimised to achieve higher yields than shake flask-based production processes. HPLC of purified BPs samples showed PHB %w/w of > 83% (Fig. 2e), which could also be improved with further downstream process optimisation. It should be noted that the purified empty BP sample gave a PHB content % w/w of about 114%, which is still considered to be within the standard deviation of PHB quantification and is likely a result of standard error but still indicates that the empty BPs are of high purity. As the overall bioprocess was not optimised, we did not consider replicates to obtain statistically significant data.

We confirmed the epitopes were presented on the BP surface and were accessible for specific antibody binding by performing ELISA using a monoclonal anti-NANP_3_ antibody as primary antibody (Fig. 2g, f). If epitopes were not BP surface exposed the primary antibody would not have bound to BPs during ELISA. B/T epitope-coated BPs bound more anti-NANP_3_ antibodies than the B-cell epitope-coated BPs. This is likely due to the T-cell epitope sequence increasing the surface exposure of the B-cell epitope.

*E. coli* contains the known endotoxin lipopolysaccharide (LPS), which induces a strong endotoxic response in humans and animals^57,58^. Hence to avoid LPS impurities that could interfere with immune responses, all BPs were produced in *Clearcoli* BL21 (DE3), which is an endotoxin-free strain that only contains a modified LPS incapable of triggering an endotoxic response in humans and animals^59^.

BPs vaccine performance was tested in sheep using an immunisation schedule that was successfully applied in previous studies using various BP vaccine candidates^33,35,38^. The B- and B/T-cell epitope-coated BPs groups showed a strong and specific immune response compared to the empty BPs and the soluble B- and B/T-cell epitope peptides (Fig. 3). The carrier control group, i.e., sheep vaccinated with empty BPs coated with only the BP anchoring protein, revealed that the empty BPs themselves are poorly immunogenic as only low anti-PhaC average EC50 titres were observed suggesting no carrier suppression occurred (Fig. 3a). This was consistent with previous studies^33,35,38^. This is likely due to empty BPs not comprising immunogenic components. For the B/T-cell epitope BP group, average EC50 titres were up to 20 times higher compared to the soluble B/T-cell epitope peptide group (Fig. 3b). For B-cell epitope BP groups, the epitope titre were up to 65 times higher compared to the soluble B-cell epitope peptide group (Fig. 3c). This boost in immune response illustrates the effectiveness of the BPs as a peptide antigen carrier. Interestingly, serum samples from sheep vaccinated with B-cell epitope-coated BPs generally showed higher overall average EC50 titres even when ELISA plates were coated with B/T-cell epitope BPs (Fig. 3). This indicates that the additional T-cell epitope is not necessary to enhance the immune response. While the empty BPs were not immunogenic themselves, they were able to efficiently deliver epitopes for specific strong induction of immune responses as reflected by the high average anti-NANP_3_ antibody EC50 titres for B-cell epitope (NANP_3_)-coated BPs and B/T-cell epitope (NANP_3_ plus T-cell epitope)-coated BPs, respectively. Comparing to the low titres induced by the linear peptide epitopes highlights the immune boosting effect of the BPs.

Immunoblots showed that specific B- and B/T-cell epitope antibodies were only induced in sheep vaccinated with B- and B/T-cell epitope-coated BPs (Fig. 4). Sheep vaccinated with empty BPs, soluble B-cell epitope peptides and B/T-cell epitope peptides did not produce any antibodies detectable by immunoblot as indicated by the absence of protein bands (Fig. 4b–d). However, sheep vaccinated with the B- and B/T-cell epitope BPs produced specific antibodies indicated by the presence of a single band corresponding to the theoretical molecular weight of the B- and B/T-cell epitope BP fusion proteins (Fig. 4e, f). This again highlights the immune boosting effect and specificity of the BPs as a peptide antigen carrier.

To determine that the antibodies bound to *P. falciparum* sporozoites, we stained with sheep serum-derived IgG as a primary antibody and a fluorescent secondary antibody and imaged them using fluorescent microscopy (Fig. 5). We demonstrated that the IgG generated by our B- and B/T-cell epitope-coated BPs bound to sporozoites as the images were comparable to the positive anti-CSP control. We then further tested these sheep antibodies for their ability to functionally inhibit traversal of sporozoites through human hepatocytes. We confirmed that the antibodies did inhibit traversal at a level comparable to the positive anti-CSP control that blocks sporozoite gliding motility (Fig. 6).

Previously mice have been vaccinated with B- and B/T-cell epitope linear and branched peptides identical to those used to coat BPs in this study. Peptides were formulated in Freund’s adjuvant and 50 µg of peptides were injected into BALB/c and C57BL/10 mice. In said study antibody titres were measured as endpoint titres defined as the last serum dilution exhibiting an optical density greater than plus 3-times standard deviations of the pre-immune sera optical density. The linear peptides induced endpoint antibody titres of 163,840 and 327,680 in BALB/c and C57BL/10 mice, respectively. The branched peptides induced endpoint antibody titres of 327,680 and 163,840 in BALB/c and C57BL/10 mice respectively^54^. Both B/T and B-cell epitope-coated BPs, respectively, developed in this study, induced endpoint titres of >409,600, which highlights the immunogenicity boosting effect of presenting these epitopes on BPs.

Several experimental malaria vaccines like the one described here have been produced in the past. One of the most promising of these is a CSP based virus-like particle, dubbed R21. In mice R21 was immunogenic at a low dose and unlike the RTS,S, but like our B- and B/T-cell epitope-coated BPs, produced a minimal response to the fusion protein partner. This vaccine has now progressed to phase I/II human clinical trials^12^. The R21 vaccine utilises the hepatitis B S protein, which self assembles into virus-like particles. The NANP3 region is fused to S protein to assemble virus-like particles presenting the NANP3 epitopes. The R21 animal study achieved maximum average NANP3 endpoint titres of ~ 512,000 in BALB/c mice. The B- and B/T-cell epitope-coated BPs outlined in this study achieved average endpoint titres of >409,600, which could be further improved by optimising dose and formulation. It is important to note that our comparable titres were achieved in large animal such as sheep, whose body weight is more like human body weight, hence these titres are more significant toward human vaccine development.

The BP-based vaccine described here was produced in *E. coli* cells, an industrial production host used for large-scale and cost-effective manufacture of biologics and purified using a simple downstream process such as cell lysis and detergent washes. Hence, it is predicted to be suitable for industrial large-scale manufacture and to be significantly more cost-effective than the R21 vaccine, which was produced in yeast. In addition, the BP vaccines are stable at ambient temperature as was previously shown^49,60^. Cost-effective scalable manufacture combined with vaccine stability will facilitate dissemination in developing countries where malaria is most relevant.

## Methods

### Biological materials

Unique biological materials described in this study that are not available from standard commercial resources are available from the authors upon request.

### Bacterial strains and growth conditions

Details of all cells and strains used in this study can be found in Supplementary Table.

### *E. coli* Top 10, a molecular cloning host, was grown in Luria Bertani (LB) media

(Difco, USA) containing ampicillin (100 µg/ml) at 37 °C. ClearColi BL21 (DE3) (Lucigen, USA), an endotoxin-free host, was engineered for BP production. For BP production 200 ml pre-culture in synthetic media was used to inoculate the 1 L main culture (synthetic media) in the Bioflo®320 (Eppendorf, Germany). This was followed by fed-batch cultivation where DO and pH were maintained for 48 h until harvesting of biomass.

### Plasmid construction

Details of all plasmids used in this study can be found in Supplementary Table 4. Cloning techniques such as plasmid preparation, restriction digests, gel purification, ligations, competent cell preparations and transformations were performed as per user manuals and as described elsewhere^61^. The DNA fragments encoding two repeats of the B-cell epitope (NANPNANPNANP) or the B/T-cell epitope with amino acid sequence (DPNANPNVDPNANPNVNANPNANPNANPEYLNKIQNSLSTEWS PCSVT) derived from circumsporozoite protein (CSP) were synthesised (Biomatik, Canada) and subcloned into plasmid pPolyN generating the final plasmids pET-14b B12N PhaC and pET-14b BT2N PhaC. The DNA sequence was confirmed by the Griffith University DNA Sequencing Facility (Griffith University, Australia). The sequence confirmed plasmids were transformed into the endotoxin-free production host, ClearColi BL21 (DE3) (Lucigen, USA) that was previously engineered for BP production^60^.

### BP purification and sterilisation

BP harbouring biomass was mechanically lysed using a microfluidizer M-110P (Microfluidics, USA). Cell lysate was centrifuged at 8000 x *g* for 30 min at 4^o^C to sediment the BPs, which were then sequentially washed 5 mM EDTA and detergent 2% (v/v) Triton X-100 containing wash buffers (10 mM Tris.HCl, pH 7.5)^38^. Purified BPs were stored in 10 mM Tris buffer (pH 7.5) containing 20% (v/v) ethanol at 4 °C for further analysis. BPs were then washed three times with (10 mM Tris.HCl, pH 7.5) before being resuspended in 10 mM Tris buffer pH 7.5, with 20% ethanol. One-hundred microlitres of the 20% (w/v) BP suspension was plated onto Thermo Scientific™ Blood Agar (TSA with Sheep Blood) (Thermo Fisher, USA) Medium to assess sterility.

### BP protein analysis

BP-associated fusion proteins were analysed by SDS-PAGE as described elsewhere^62^. Briefly, protein samples were separated in 10% (vol/vol) polyacrylamide separating gels with 4% (vol/vol) polyacrylamide stacking gels at 170 V for 45 min. The molecular mass of the samples was estimated using GangNam-STAIN prestained protein ladder (iNtRON Biotechnology) or Novex™ Sharp Pre-stained Protein Standard (Invitrogen). SDS-PAGE were stained by incubating overnight in Staining solution (0.05% (wt/vol) coomassie brilliant blue R-250 dye, 50% (vol/vol) ethanol, and 10% (vol/vol) acetic acid), then rinsed and destained in 50% (vol/vol) ethanol and 10% (vol/vol) acetic acid for 6 h. Images were taken using a BIO-RAD Gel Doc XR + with the Image Lab^TM^ Software (Bio-Rad).

### Protein quantification

Protein concentration and purity was determined via densitometry on SDS-PAGE using Image J version 1.52a (National Institute of Health). Bands were compared to a standard curve prepared from known concentrations of bovine serum albumin (BSA) standard as described elsewhere^47^. Additionally, protein concentration was also determined using the Quick Start^TM^ Bradford 1x Dye Reagent (Bio-Rad) as per manufacturer’s instructions. Absorbance at 595 nm was measured using the Biotek Synergy 2 microplate reader (Biotek). Binding and purification capacity were calculated as μg of protein per mg of BP. Cleavage efficiency was calculated by dividing the amount of purified protein by the amount protein bound and multiplying by 100.

### Mass spectroscopy quad time of flight (MALDI-TOF) analysis

Purified protein bands from the SDS-PAGE gel were excised and subjected to tryptic hydrolysis as described^63^. The resulting extracted tryptic peptide samples were then analysed by matrix-assisted laser desorption ionisation time-of-flight/mass spectrometry (MALDI-TOF/MS).

### Transmission electron microscope (TEM)

BPs were processed and sectioned for TEM. Briefly, the samples were fixed in 2.5% glutaraldehyde, and embedded in 2% agarose. All subsequent processing was done in a Biowave processing microwave (PELCO). The samples were post fixed with 1% osmium tetroxide with 4 times 2 min on/off cycles at 80 W. They were washed twice in water at 80 W 40 s with vacuum. Dehydration through a graded series of ethanols, 30, 50, 70, 90, 2 × 100% all at 150 W for 40 s with vacuum. The samples were then infiltrated with Epon resin using graded mixes with ethanol, 3:1, 1:1, 1:3 then 2 times 100% resin all at 250 W for 3 min with vacuum. Samples were polymerised at 60 °C for 2 days. Ultrathin sections of 60 nm were cut using a diamond knife on an Ultracut UC6 ultramicrotome (Leica) and mounted on Formvar coated copper grids. The sections were stained with 5% uranyl acetate in 50% ethanol 5 min then rinsed in water and stained again with Reynolds Lead Citrate for 3 min and rinsed 21 in water again before being air dried. TEM micrographs of the sectioned samples were imaged using a Hitachi HT7700 (Hitachi) at 80 kV.

### Size and zeta potential measurements

BP particle size was determined by dynamic light scattering (DLS) analysis using the Litesizer 500(Anton Paar) at room temperature (25 °C). Zeta potential of the BP was determined by electrophoretic light scattering coupled with phase analysis light scattering using Litesizer (Anton Paar). All BP samples were measured in 0.1% (wt/vol) of the wet particles in 50 mM Tris-HCl, pH 7.5, and the soluble protein samples were measured in 50 mM Tris-HCl, pH 7.5. Three technical replicates were performed.

### PHB content analysis

Analysis of cellular PHB content and purified BP samples was based on the method of Karr et al.^64^. Twenty-five micolitre samples of culture were pelleted by immediate centrifugation (5000 ×  *g* for 10 min at 4 °C), rinsed with 5 ml of milli-Q water, and centrifuged again. Pellets were stored at −80 °C until further analysis. Pellets were then freeze-dried and weighed. Next, 2 ml of concentrated sulphuric acid was added to the pellet and PHB was acid hydrolysed to crotonic acid for 1 h at 90 °C. The samples were filtered using a 0.22 μm filter (Merck Millipore) and diluted 1 in 500. Adipic acid was added in 1:1 to the sample (400 mg/L final concentration) and used as an internal standard in HPLC analysis. For this, 30 μL of sample was injected into an Agilent Hiplex H column (300 × 7.7 mm, PL1170–6830) with a guard cartridge (Security Guard Carbo-H, 4 × 3 mm, Phenomenex PN: AJO-4490) using an Agilent 1200 HPLC system equipped with high-performance autosampler (Agilent HiP-ALSSL, G1367C), binary pump (Agilent Bin Pump, G1312A), degasser (Agilent Degasser, G1379B), thermostatted column compartment (Agilent TCC, G1316B), multiwavelength (Agilent MWD, G1365B) and refractive index (Agilent RID, G1362A) detectors. Analytes were eluted with 4 mM sulphuric acid at 0.6 ml/min flow rate, column temperature of 65 °C, and monitored at 210 nm UV and positive polarity at 40 °C on the RID, over an isocratic run of 30 min. Crotonic acid was used as the standard for quantification. Peak areas were integrated using ChemStation (Rev B.03.02 [341]). The PHB % w/w was calculated using the determined crotonic acid concentration in the sample (based on Karr et al.)^64^ and the analysed samples dry weight.

### Vaccine formulation and immunogenicity study

Vaccine formulations contained either empty BPs, B-cell epitope peptide, B/T-cell epitope peptide, B-cell epitope-coated BPs or B/T-cell epitope-coated BPs. B- and B/T-cell epitope peptides were ordered from GenScript. All vaccines were formulated to contain 55.6% v/v of the adjuvant Xstend III and B- and B/T-cell epitope doses of 25 µg and 50 µg per injection, respectively. The empty BP group was formulated so that the amount of PhaC injected would equal the maximum amount of PhaC for any other group. The groups were double blinded, and injections were given to eight female Merino sheep per group on three occasions (days 0, 14 and 28), serum samples were taken on day 0, 14, 21, 28, 42, and 56 by jugular vein venipuncture. Approximately 18–20 ml serum was collected from each sheep, which was processed within 4 h of collection by accredited or trained personnel. Samples were aliquoted into cryovials labelled with a unique specimen number and stored in dry ice or at −80 ^o^C until needed. This study protocol received ethical approval (approval numbers ELA1800507 and ELA1900168) from the Animal Research Authority of the Yarrandoo Elanco Animal Ethics Committee. This study complied with all relevant ethical regulations for animal testing and research.

### Enzyme-linked immunosorbent assay (ELISA)

Serum antibody responses were analysed by ELISA. High-binding plates (Greiner Bio-One, Germany) were coated overnight at 4 °C with 100 µl of 5 µg/ml B-cell epitope- or B/T-cell epitope-coated BPs in phosphate-buffered saline containing 0.05% (v/v) Tween 20, pH 7.5 (PBST). As controls, plates were also coated overnight at 4 °C with corresponding amounts of empty BPs in PBST or only PBST. Plates were washed three times with PBST using the ELx 405 Select cw plate washer (BioTek, USA) and blocked with 3% (w/v) BSA for 1 h at 25 °C. Plates were washed with PBST three times again and incubated with primary polyclonal antibodies, sera taken from individual sheep, diluted with PBST at concentrations ranging from 1/400 to 1/409,600 at 25 °C for 1 h. When analysing antibody responses against empty BPs, primary antibody dilutions ranged from 1/400 to 1/1600. Additional control ELISAs to assess BP surface exposure of epitopes were performed using monoclonal anti-NANP3 antibodies (clone 2A10, MRA-183A, BEI resources) as the primary antibody instead of sheep sera at the same dilutions and using Rabbit Anti-Mouse IgG H&L (HRP) (Abcam, UK, Catalogue No.ab6728) diluted 1:1000 as secondary antibody. After three more washes with PBST, plates were incubated with secondary HRP-conjugated antibodies, Rabbit Anti-Sheep IgG H&L (HRP) (Abcam, UK, Catalogue No. ab6747), diluted 1/20,000 with PBST and incubated for 1 h at 25 °C to detect bound sheep antibodies. After washing, o-phenylenediamine substrate (Abbott Diagnostics, IL, USA) was added at a concentration of 0.7 mg/ml in Stable Peroxide buffer (Thermo Fisher, USA) on plates and incubated for 15 min at 25 °C. The reaction was stopped by adding 50 µL of 0.5 N H_2_SO_4_, and the results were measured at 490 nm on the Synergy™ 2 Multi-Mode Microplate Reader (BioTek, USA). The blank values were subtracted, and the values converted to EC_50_, which gave half the maximum response and absorbance (mean values ± SEM, *n* = 6) and estimated by a sigmoid curve fitted with a straight line (*y* = *mx* + *c*) using linear interpolation.

Minitab 19 (Minitab, State, College, PA, USA) was used for statistical analysis of data. Statistical differences between two groups were determined with a nonparametric Mann–Whitney test. Each data point represented the mean of eight sheep ± the standard error. Statistical significance was determined when *p* < 0.05.

### Immunoblotting

To investigate the specificity of the IgG response, pooled sera from sheep immunised with soluble B-cell epitope, soluble B/T-cell epitope, empty BPs, B-cell epitope-coated BPs or B/T-cell epitope-coated BPs were diluted 10,000-fold and used for immunoblotting against *E. coli* whole-cell lysate containing various BPs and enriched BPs after they were transferred from Bis-Tris gel to nitrocellulose membranes (Life Technology, USA). *E. coli* whole-cell lysate not producing any fusion protein, i.e., no BPs, served as negative control. Rabbit Anti-Sheep IgG H&L (HRP) (Abcam, UK, Catalogue No. ab6747) was diluted 20,000-fold and used for detection of bound IgG antibodies.

Signal was developed by incubating the membrane with SuperSignal West Pico StablePeroxide Solution and SuperSignal West Pico Luminol/Enhancer Solution (Thermo Scientific, USA) for 5 min. The image was then captured with the ODYSSEY Fc imaging system (LI-COR, USA).

### IgG purification

IgG was purified from pooled sheep serum using our previously developed BPs coated with the protein-G IgG-binding domain (GB1)_3_^52^. BPs were centrifuged at 6000 x *g* for 4 m before being washed twice by resuspending in PBS buffer. BPs were then resuspended to 5% w/v using day 56 pooled sheep serum and incubated at 25 ^o^C for 30 min at 200 rpm. BPs were then centrifuged and washed three times with PBS buffer. To release pure IgG, BPs were resuspended to 5% w/v in 50 mM glycine (pH2.7) and incubated at 25 ^o^C for 5 min at 200 rpm. BPs were then centrifuged at 15,000 x *g* for 4 min to separate soluble IgG. In all, 20 mM K_2_HPO_4_ was added to neutralise the solution. Samples were analysed by SDS-PAGE to confirm IgG presence and stored at −20 ^o^C.

### Parasite maintenance

*P. falciparum* NF54 asexual stages were maintained in human type O-positive erythrocytes (Melbourne Red Cross) in RPMI-HEPES supplemented with 10% heat-inactivated human serum (Melbourne Red Cross) at 37 °C. Gametocytes for transmission to mosquitoes on day 17 were generated using the “crash” method with daily media changes^65^.

### Mosquito infection and parasite development

Five to 7-day-old female *Anopheles stephensi* mosquitoes were fed on asynchronous gametocytes, diluted to 0.3%–0.6% stage V gametocytemia. Mosquitoes were sugar starved for 2 days after blood meal to select for blood-fed mosquitoes. Surviving mosquitoes were provided 5% glucose ad libitum via filter paper wicks or sugar cubes. Oocyst numbers were enumerated from midguts dissected from cold-anaesthetised and ethanol-killed mosquitoes 7 days post-infection and stained with 0.1% mercurochrome. Salivary glands were dissected from mosquitoes (days 16–20 after blood meal), crushed using a pestle, and glass wool filtered to obtain sporozoites.

### Sporozoite staining

Salivary gland sporozoites were fixed in 4% paraformaldehyde solution for 20 min at room temperature (RT), washed and dried on glass slides. Samples were blocked in 2% BSA, 0.1% Triton-X-100 in PBS for 1 h at room temp (RT). Primary antibodies were incubated at 10 µg/ml in blocking buffer for 1 h at RT. Samples were washed three times with PBS and incubated in secondary anti-sheep (AF488 donkey anti-sheep IgG: (CiteAb, Catalogue No. A11015)) or anti-mouse (AF488 donkey anti-mouse IgG: (CiteAb, Catalogue No. A11029)) Alexa 488 antibodies (1:500 dilution) at RT before washing three times in PBS, staining for 5 min in 2 µg/ml DAPI, washing three times and air-drying. Slides were mounted beneath cover glasses with Prolong gold antifade reagent. All micrographs were acquired on a Zeiss LSM880 confocal microscope with Airyscan, processed with ImageJ and are presented with identical scaling.

### Hepatocyte cell traversal assay with sporozoites

Human hepatoblastoma cells (HC-04 hepatocytes)^66^ were maintained on Iscove’s Modified Dulbecco’s Media (IMDM) and supplemented with 5% heat-inactivated foetal bovine serum (FBS) at 37^ o^C in 5% CO_2_. Cells were split 1:6 every 2–3 days once they reached 90% confluency. The traversal activity of sporozoites was measured using a standard cell-wounding assay^53^. In brief, 1 × 10^5^ HC-04 cells were seeded into wells of a 96-well plate (Corning, Sigma-Aldrich). The following day, wells were seeded with 3 × 10^4^ sporozoites (MOI 0.3) for 3 h in the presence of 1 mg/ml FITC-labelled dextran (10,000 molecular weight [MW]; Sigma-Aldrich). Cells were washed to remove sporozoites, trypsinized to obtain a single-cell suspension and FITC-dextran positive cells enumerated by flow cytometry. Antibodies were incubated at 10 µg/ml. For each condition, triplicate samples of 10,000 cells were counted by FACS in each of the three independent experiments and data statistically analysed using Graphpad Prism 7. An exemplification of the gating strategy is provided in the Supplementary Information (Supplementary Fig. 1).

### Reporting summary

Further information on research design is available in the Nature Research Reporting Summary linked to this article.

## Supplementary information

Supplementary Information

Reporting Summary

## Data Availability

All data from this study are available from the corresponding author upon reasonable request. Plasmids newly generated in this study are available from Addgene with ID numbers 177646 and 177647.

## Code availability

No custom computer code or mathematical algorithm that is deemed central to the conclusions was used in this study.
