# Supplementary material for: Epitope-coated polymer particles elicit neutralising antibodies against Plasmodium falciparum sporozoites
Source: NPJ Vaccines. 2021 Nov 29;6:141. doi: 10.1038/s41541-021-00408-2 (PMC8630014; doi:10.1038/s41541-021-00408-2)
Supplement: Supplementary file 1 — Supplementary Information [file 41541_2021_408_MOESM1_ESM.pdf]

Supplementary Material

Epitope-coated polymer particles elicit neutralizing antibodies against *Plasmodium falciparum* sporozoites

Ben Evert[1], Shuxiong Chen[1], Julie Healer[2], Alan Cowman[2], Ryan Steel[2], Robyn McConville[2], Justin Boddey[2], Lucas Huntimer[3] and Bernd H.A. Rehm[1,4]*,

[1]Centre for Cell Factories and Biopolymers, Griffith Institute for Drug Discovery, Griffith University, Brisbane, Australia.

[2]Walter and Eliza Hall Institute of Medical Research, Melbourne, Australia

[3]Elanco, Indianapolis, USA

[4]Menzies Health Institute Queensland (MHIQ), Griffith University, Gold Coast, Australia.

Supplementary Table 1. Sequences of proteins used in this study.

Fusion Protein	Sequence	Size
PhaC	MATGKGAAASTQEGKSQPFKVTPGPFDPATWLEWSRQWQGTEG<br>NGHAAASGIPGLDALAGVKIAPAQLGDIQQRYMKDFSALWQAMAEG<br>KAEATGPLHDRRFAGDAWRTNLPYRFAAAFYLLNARALTELADAVE<br>ADAKTRQRIRFAISQWVDAMSPANFLATNPEAQRLLIESGGESLRA<br>GVRNMMEDLTRGKISQTDESAFEVGRNVAVTEGAVVFENEYFQLL<br>QYKPLTDKVHARPLLMVPPCINKYYILDLQPESSLVRHVVEQGHTVF<br>LVSWRNPDASMAGSTWDDYIEHAAIRAIEVARDISGQDKINVLGFCV<br>GGTIVSTALAVLAARGEHPAASVTLLTTLLDFADTGILDVFVDEGHVQ<br>LREATLGGGAGAPCALLRGLELANTFSFLRPNDLVWNYVVDNYLKG<br>NTPVPFDLLFWNGDATNLPGPWYCWYLRHTYLQNELKVPGKLTVC<br>GVPVDLASIDVPTYIYGSREDHIVPWTAAYASTALLANKLRFVLGAS<br>GHIAGVINPPAKNKRSHWTNDALPESPQQWLAGAIEHHGSWWPD<br>WTAWLAGQAGAKRAAPANYGNARYRAIEPAPGRYVKAKA	64.33 kDa
B cell epitope-PhaC	MNANPNANPNANPNANPNANPNANPNANP-Linker-ATGKGAAASTQEG<br>KSQPFKVTPGPFDPATWLEWSRQWQGTEGNGHAAASGIPGLDAL<br>AGVKIAPAQLGDIQQRYMKDFSALWQAMAEGKAEATGPLHDRRFA<br>GDAWRTNLPYRFAAAFYLLNARALTELADAVEADAKTRQRIRFAISQ<br>WVDAMSPANFLATNPEAQRLLIESGGESLRAGVRNMMEDLTRGKIS<br>QTDESAFEVGRNVAVTEGAVVFENEYFQLLQYKPLTDKVHARPLLM<br>VPPCINKYYILDLQPESSLVRHVVEQGHTVFLVSWRNPDASMAGST<br>WDDYIEHAAIRAIEVARDISGQDKINVLGFCVGGTIVSTALAVLAARG<br>EHPAASVTLLTTLLDFADTGILDVFVDEGHVQLREATLGGGAGAPCA<br>LLRGLELANTFSFLRPNDLVWNYVVDNYLKGNTPVPFDLLFWNGDA<br>TNLPGPWYCWYLRHTYLQNELKVPGKLTVCGVPVDLASIDVPTYIY<br>GSREDHIVPWTAAYASTALLANKLRFVLGASGHIAGVINPPAKNKRS<br>HWTNDALPESPQQWLAGAIEHHGSWWPDWTAWLAGQAGAKRAA<br>PANYGNARYRAIEPAPGRYVKAKA	67.07 kDa
B/T cell epitope-PhaC	MDPNANPNVDPNANPNVNANPNANPNANPEYLNKIQNSLSTEWSP<br>CSVTDPNANPNVDPNANPNVNANPNANPNANPEYLNKIQNSLSTE<br>WSPCSVT-Linker-ATGKGAAASTQEGKSQPFKVTPGPFDPATWLE<br>WSRQWQGTEGNGHAAASGIPGLDALAGVKIAPAQLGDIQQRYMKD<br>FSALWQAMAEGKAEATGPLHDRRFAGDAWRTNLPYRFAAAFYLLN<br>ARALTELADAVEADAKTRQRIRFAISQWVDAMSPANFLATNPEAQR<br>LLIESGGESLRAGVRNMMEDLTRGKISQTDESAFEVGRNVAVTEGA<br>VVFENEYFQLLQYKPLTDKVHARPLLMVPPCINKYYILDLQPESSLV<br>RHVVEQGHTVFLVSWRNPDASMAGSTWDDYIEHAAIRAIEVARDIS<br>GQDKINVLGFCVGGTIVSTALAVLAARGEHPAASVTLLTTLLDFADT<br>GILDVFVDEGHVQLREATLGGGAGAPCALLRGLELANTFSFLRPND<br>LVWNYVVDNYLKGNTPVPFDL<br>LFWNGDATNLPGPWYCWYLRHTYLQNELKVPGKLTVCGVPVDLAS<br>IDVPTYIYGSREDHIVPWTAAYASTALLANKLRFVLGASGHIAGVINP<br>PAKNKRSHWTNDALPESPQQWLAGAIEHHGSWWPDWTAWLAGQ<br>AGAKRAAPANYGNARYRAIEPAPGRYVKAKA	74.92 kDa
B cell epitope peptide	MNANPNANPNANPNANPNANPNANPNANP	2.53 kDa
B/T cell epitope peptide	MDPNANPNVDPNANPNVNANPNANPNANPEYLNKIQNSLSTEWSP<br>CSVTDPNANPNVDPNANPNVNANPNANPNANPEYLNKIQNSLSTE<br>WSPCSVT	10.25 kDa

Supplementary Table 2. Identification of proteins using tryptic peptide fingerprinting applying MALDI-TOF/MS.

Identified peptides are highlighted in green.

Sample Name	Identified Peptides
B cell epitope-PhaC fusion protein	**B cell epitope:**<br>MNANPNANPNANPGPGPGNANPNANPNANP<br><br>**PhaC:**<br>ATGKGAAASTQEGKSQPFKVTPGPFDPATWLEWSRQWQGTEGNGHAAA<br>SGIPGLDALAGVK**IAPAQLGDIQQR**YMKDFSALWQAMAEGK**AEATGPLHD**<br>**RRFAGDAWRTNLPYRFAAAFYLLNAR**ALTELADAVEADAKTRQRIRFAISQ<br>WVDAMSPANFLATNPEAQR**LLIESGGESLR**AGVRNMMEDLTRGK**ISQTDE**<br>**SAFEVGR**NVAVTEGAVVFENEYFQLLQYKPLTDKVHARPLLMVPPCINK**YY**<br>**ILDLQPESSLVRHVVEQGHTVFLVSWR**NPDASMAGSTWDDYIEHAAIRAIE<br>VARDISGQDKINVLGFCVGGTIVSTALAVLAARGEHPAASVTLLTTLLDFADT<br>GILDVFVDEGHVQLR**EATLGGGAGAPCALLR**GLELANTFSFLRPNDLVWN<br>YVVDNYLKGNTPVPFDLLFWNGDATNLPGPWYCWYLRHTYLQNELKVPG<br>K**LTVCGVPVDLASIDVPTYIYGSR**EDHIVPWTAAYASTALLANKLRFVLGAS<br>GHIAGVINPPAKNKRSHWTNDALPESPQQWLAGAIEHHGSWWPDWTAWL<br>AGQAGAKR**AAPANYGNAR**YR**AIEPAPGR**YVKAKA
B/T cell epitope-PhaC fusion protein	**B/T cell epitope:**<br>MDPNANPNVDPNANPNVNANPNANPNANPEYLNKIQNSLSTEWSPCSVT<br>GPGP**GDPNANPNVD**PNANPNVNANPNANPNANPEYLNKIQNSLSTEWSP<br>CSVT<br>**PhaC:**<br>ATGKGAAASTQEGKSQPFKVTPGPFDPATWLEWSRQWQGTEGNGHAAA<br>SGIPGLDAL**AGVKIAPAQLGDIQQRY**MKDFSALWQAMAEGKAEATGPLHD<br>RRF**AGDAWRTNLPYRFAAAFY**LLNARALTELADAVEADAKTRQRIRFAISQ<br>WVDAMSPANF**LATNPEAQRLL**IESGGESLRAGVRNMMEDL**TRGKISQTDE**<br>**SAFEVGRNVAVTEGAVVF**ENEYFQLLQYKPLTDKVHARPLLMVPPCINKYY<br>ILDLQPESSL**VRHVVEQGHTVF**LVSWRNPDASMAGSTWDDYIEHAAIR**AIE**<br>**VARDISGQDKINVL**GFCVGGTIVSTALAVL**AARGEHPAASVTLL**TTLLDFAD<br>TGILDVFVDEGHVQLREATLGGGAGAPCALL**RGLELANTFSFLRPNDLVW**<br>NYVVDNYLKGNTPVPFDLLFWNGDATNLPGPWYCW**YLRHTY**LQNELKVP<br>GKLTVCGVPVDLASIDVPTYIY**GSREDHIVPW**TAAYASTALLANKLRFVLGA<br>SGHIAGVINPPAKNKRSHWTNDALPESPQQWLAGAIEHHGSWWPDWTAW<br>**LAGQAGAKRAAPANY**GNARY**RAIEPAPGRY**VKAKA

Supplementary Table 3. Strains used in this study.

Isolate	Use	Notable features	Reference
E. coli Top10	Plasmid propagation		Invitrogen, Carlsbad, CA, United States
E. coli Clearcoli	PHB bead production	Containing pMCS69 (Cm^R). Induction via 1mM IPTG	[1]
P. falciparum NF54	Sporozoite staining and traversal assay		[2]
HC-04 hepatocytes	Cell traversal assay		[3]

Supplementary Table 4. Plasmids used in this study.

DNA	Use	Notable features	Reference
pET14b-phaC	Encodes PhaC for PHB production	phaC (64.33 kDa) under T7 promoter, Amp 100	[4]
pMCS69	Encodes PhaA and PhaB for PHB production	phaA, phaB under CAT promoter, Cm 50	[4]
pET14b B cell epitope-PhaC	Encodes PHB synthase fusion with B cell epitopes	B cell epitope-PhaC (64.33 kDa) under T7 promoter, Amp 100	This study
pET14b B/T cell epitope-PhaC	Encodes PHB synthase fusion with B/T cell epitopes	B/T cell epitope-PhaC (74.92 kDa) under T7 promoter, Amp 100	This study
pET14b PhaC-(GB1)3	Encodes PHB synthase fusion with protein G binding domain	PhaC-(GB1)3 under T7 promoter, Amp 100	[5]

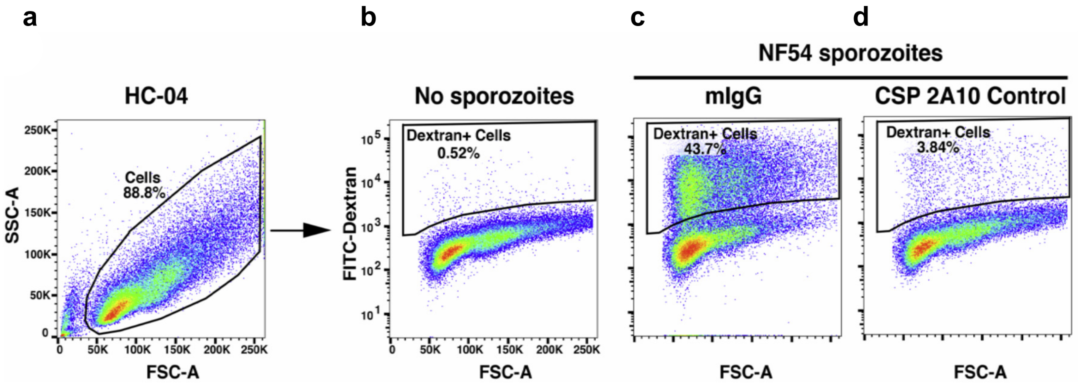

Supplementary Figure 1. Gating strategy used in FACS analysis to generate traversal inhibition data shown in Figure 6. FACS dot plots measuring FITC-dextran uptake into human HC-04 hepatocytes following incubation with sporozoites (traversal). a, shows gating for HC-04 cells. b, shows no sporozoite control. c, shows addition of sporozoites in the presence of naïve mouse IgG (10 μg/ml). d, shows addition of sporozoites in the presence of mouse anti-CSP 2A10 control antibodies (monoclonal anti-NANP3 antibodies). Shown is a single representative of three experiments. The same gating strategy was previously used to assess cell traversal by *P. falciparum* (see Figures 4 in Yang et al [6,7]).

References

1 Amara, A. A. & Rehm, B. H. Replacement of the catalytic nucleophile cysteine-296 by serine in class II polyhydroxyalkanoate synthase from *Pseudomonas aeruginosa*-mediated synthesis of a new polyester: identification of catalytic residues. *Biochem J* **374**, 413-421, doi:10.1042/BJ20030431 (2003).

2 Ponnudurai, T., Leeuwenberg, A. D. & Meuwissen, J. H. Chloroquine sensitivity of isolates of *Plasmodium falciparum* adapted to in vitro culture. *Trop Geogr Med* **33**, 50-54 (1981).

3 Sattabongkot, J. *et al.* Establishment of a human hepatocyte line that supports in vitro development of the exo-erythrocytic stages of the Malaria parasites *Plasmodium falciparum* and P. vivax. *Am J Trop Med Hyg* **74**, 708-715, doi:DOI 10.4269/ajtmh.2006.74.708 (2006).

4 Jahns, A. C., Haverkamp, R. G. & Rehm, B. H. Multifunctional inorganic-binding beads self-assembled inside engineered bacteria. *Bioconjug Chem* **19**, 2072-2080, doi:10.1021/bc8001979 (2008).

5 Jahns, A. C. *et al.* In vivo self-assembly of fluorescent protein microparticles displaying specific binding domains. *Bioconjug Chem* **24**, 1314-1323, doi:10.1021/bc300551j (2013).

6 Yang, A.S.P. *et al.* AMA1 and MAEBL are important for *Plasmodium falciparum* sporozoite infection of the liver. Cell. Microbiol. e12745 (2017) https://doi.org/10.1111/cmi.12745

7 Yang, A.S.P. *et al.* Cell Traversal Activity Is Important for *Plasmodium falciparum* Liver Infection in Humanized Mice. Cell Rep.18, 3105-3116 (2017) https://doi.org/10.1016/j.celrep.2017.03.017
