# Supplementary material for: Epitope-coated polymer particles elicit neutralising antibodies against Plasmodium falciparum sporozoites
Source: NPJ Vaccines. 2021 Nov 29;6:141. doi: 10.1038/s41541-021-00408-2 (PMC8630014; doi:10.1038/s41541-021-00408-2)
Supplement: Supplementary file 2 — Reporting Summary [file 41541_2021_408_MOESM2_ESM.pdf]

nature portfolio

Corresponding author(s): Bernd Rehm

Last updated by author(s): Sep 10, 2021

Reporting Summary

Nature Portfolio wishes to improve the reproducibility of the work that we publish. This form provides structure for consistency and transparency in reporting. For further information on Nature Portfolio policies, see our Editorial Policies and the Editorial Policy Checklist.

Statistics

For all statistical analyses, confirm that the following items are present in the figure legend, table legend, main text, or Methods section.

n/a	Confirmed	
☐	☒	The exact sample size (n) for each experimental group/condition, given as a discrete number and unit of measurement
☐	☒	A statement on whether measurements were taken from distinct samples or whether the same sample was measured repeatedly
☐	☒	The statistical test(s) used AND whether they are one- or two-sided<br>*Only common tests should be described solely by name; describe more complex techniques in the Methods section.*
☒	☐	A description of all covariates tested
☒	☐	A description of any assumptions or corrections, such as tests of normality and adjustment for multiple comparisons
☐	☒	A full description of the statistical parameters including central tendency (e.g. means) or other basic estimates (e.g. regression coefficient) AND variation (e.g. standard deviation) or associated estimates of uncertainty (e.g. confidence intervals)
☒	☐	For null hypothesis testing, the test statistic (e.g. F, t, r) with confidence intervals, effect sizes, degrees of freedom and P value noted<br>*Give P values as exact values whenever suitable.*
☒	☐	For Bayesian analysis, information on the choice of priors and Markov chain Monte Carlo settings
☒	☐	For hierarchical and complex designs, identification of the appropriate level for tests and full reporting of outcomes
☒	☐	Estimates of effect sizes (e.g. Cohen's d, Pearson's r), indicating how they were calculated

Our web collection on statistics for biologists contains articles on many of the points above.

Software and code

Policy information about availability of computer code

Data collection	No software was used.
Data analysis	Minitab 19 and Graphpad Prism 7 was used for statistical analysis of data.

For manuscripts utilizing custom algorithms or software that are central to the research but not yet described in published literature, software must be made available to editors and reviewers. We strongly encourage code deposition in a community repository (e.g. GitHub). See the Nature Portfolio guidelines for submitting code & software for further information.

Data

Policy information about availability of data

All manuscripts must include a data availability statement. This statement should provide the following information, where applicable:

- Accession codes, unique identifiers, or web links for publicly available datasets
- A description of any restrictions on data availability
- For clinical datasets or third party data, please ensure that the statement adheres to our policy

All data from this study are available from the corresponding author upon reasonable request.

Field-specific reporting

Please select the one below that is the best fit for your research. If you are not sure, read the appropriate sections before making your selection.

☒ Life sciences ☐ Behavioural & social sciences ☐ Ecological, evolutionary & environmental sciences

For a reference copy of the document with all sections, see nature.com/documents/nr-reporting-summary-flat.pdf

Life sciences study design

All studies must disclose on these points even when the disclosure is negative.

Sample size	The animal trial group sizes were approved by the animal ethics committee applying power calculations using One-Way ANOVA to calculate the minimal number of animals required to reach a significant result.
Data exclusions	No data were excluded.
Replication	Power calculations were used to inform the number of replicates where significance of data differences were deemed to be critical for conclusions. A minimum of three replicates was applied for experiments where reproducibility was needed to support conclusions.
Randomization	This was not relevant to this study as number of replicates and multiple controls supported generation of conclusive results.
Blinding	The animal study was double-blinded i.e. in the animal trial the identity of vaccines/controls was not known as well as when serum samples were analyzed it was not known with which vaccine/controls the respective animals had been vaccinated.

Reporting for specific materials, systems and methods

We require information from authors about some types of materials, experimental systems and methods used in many studies. Here, indicate whether each material, system or method listed is relevant to your study. If you are not sure if a list item applies to your research, read the appropriate section before selecting a response.

Materials & experimental systems

n/a	Involved in the study	
☐	☒	Antibodies
☒	☐	Eukaryotic cell lines
☒	☐	Palaeontology and archaeology
☐	☒	Animals and other organisms
☒	☐	Human research participants
☒	☐	Clinical data
☒	☐	Dual use research of concern

Methods

n/a	Involved in the study	
☒	☐	ChIP-seq
☐	☒	Flow cytometry
☒	☐	MRI-based neuroimaging

Antibodies

Antibodies used	ELISA: Rabbit Anti-Sheep IgG H&L (HRP) (Abcam, UK, Catalog No. ab6747); Rabbit Anti-Mouse IgG H&L (HRP) (Abcam, UK, Catalog No.ab6728); monoclonal anti-NANP3 antibody (clone 2A10, BEI resources, Catalog No. MRA-183A). FACS: monoclonal anti-NANP3 antibody (clone 2A10, BEI resources, Catalog No. MRA-183A); AF488 donkey anti sheep IgG: (CiteAb, Catalog No. A11015); AF488 donkey anti mouse IgG: (CiteAb, Catalog No. A11029)
Validation	The rabbit anti-sheep IgG H&L (HRP) and rabbit anti-mouse IgG H&L (HRP) were provided with a certificate of analysis from the supplier (see https://www.abcam.com/rabbit-sheep-igg-hl-hrp-ab6747.html and https://www.abcam.com/rabbit-mouse-igg-hl-hrp-ab6728.html). The monoclonal anti-NANP3 antibody was provided with a product information sheet by BEI resources and in our study tested against the NANP3 peptide.

Animals and other organisms

Policy information about studies involving animals; ARRIVE guidelines recommended for reporting animal research

Laboratory animals	Female Merino sheep
Wild animals	The study did not involve wild animals.
Field-collected samples	The study did not involve field-collected samples.
Ethics oversight	This study protocol was conducted under an Animal Research Authority issued by the Yarrandoo Elanco Animal Ethics Committee.

Note that full information on the approval of the study protocol must also be provided in the manuscript.

Flow Cytometry

Plots

Confirm that:

☒ The axis labels state the marker and fluorochrome used (e.g. CD4-FITC).

☒ The axis scales are clearly visible. Include numbers along axes only for bottom left plot of group (a 'group' is an analysis of identical markers).

☒ All plots are contour plots with outliers or pseudocolor plots.

☒ A numerical value for number of cells or percentage (with statistics) is provided.

Methodology

Sample preparation	Human hepatoblastoma cells (HC-04 hepatocytes) were maintained on Iscove's Modified Dulbecco's Media (IMDM) supplemented with 5% heat-inactivated fetal bovine serum (FBS) at 37 degrees in 5% CO2. Cells were split 1:6 every 2–3 days once they reached 90% confluency. 1 × 10^5 HC-04 were seeded into each well of a 96-well plate. The next day NF54 wild type Plasmodium falciparum sporozoites were dissected from the salivary glands of female Anopheles stephensi mosquitoes on day 17 post feed. Sheep antibodies, 2A10 monoclonal antibody or mouse IgG control antibody were added to freshly isolated sporozoites at a concentration of 10 ug/ml before addition of 3 × 10^4 sporozoites to each well of HC-04 cells (MOI 0.3). Antibodies were present throughout the traversal assay. HC-04 hepatocytes were trypsinized to obtain a single-cell suspension for analysis by flow cytometry.
Instrument	BD LSRFortessa X-20 Cell Analyzer, model number: H65767501001
Software	BD FACSdiva software was used to collect the flow cytometry data, and data analysed using FlowJo software for Mac version 10.8.
Cell population abundance	For each condition, triplicate samples of 10,000 cells were counted by FACS in each of the three independent experiments. Dextran positive cells were readily detected in the positive control samples, with incidence rates generally between four and fifteen percent.
Gating strategy	Cells were identified based on forward and side scatter properties, then dextran positive cells were determined based on a negative control which contained HC-04 hepatocytes in the presence of dextran without sporozoites.

☒ Tick this box to confirm that a figure exemplifying the gating strategy is provided in the Supplementary Information.
